# Efficacy of robot‐assisted minimally invasive stereotactic puncture therapy for supratentorial hypertensive intracerebral hemorrhage

**DOI:** 10.1002/brb3.3402

**Published:** 2024-02-04

**Authors:** Weiyi Han, Aotan Xie, Taoli Chen, Xiao Sun, Xianzhi Liu

**Affiliations:** ^1^ The First Affiliated Hospital of Zhengzhou University Zhengzhou Henan Province China; ^2^ Nanyang Central Hospital Nanyang Henan Province China; ^3^ The First Affiliated Hospital of Hebei North University Zhangjiakou Hebei Province China; ^4^ Nanyang Second General Hospital Nanyang Henan Province China

**Keywords:** craniotomy, intracerebral hemorrhage, robot, stereotactic

## Abstract

**Objective:**

To evaluate the efficacy of robot‐assisted minimally invasive stereotactic puncture therapy (MISPT) for supratentorial hypertensive intracerebral hemorrhage (HICH).

**Methods:**

We retrospectively analyzed 133 patients with supratentorial HICH treated using robot‐assisted MISPT (RM group; *n* = 77) or conventional craniotomy (CC group; *n* = 56). In our primary analysis, we evaluated the hematoma volume at discharge. In our secondary analyses, we evaluated the Glasgow Coma Scale (GCS) score at discharge; the operation time, intraparenchymal drainage catheter removal time, the length of hospital stay, and complications during hospitalization; the early and 6‐month postoperative mortality; and functional scores 6‐month postoperatively.

**Results:**

There were no statistical differences in the preoperative characteristics between the groups, such as age distribution (52.8 ± 9.6 vs. 55.3 ± 7.8 years), hematoma volume (38.4 ± 10.4 vs. 41.1 ± 11.0 mL), and GCS score (10.7 ± 2.2 vs. 9.8 ± 2.8). Hematoma volume at discharge did not significantly differ between the groups (2.6 ± 2.1 mL vs. 2.4 ± 2.1 mL). The GCS score at discharge was significantly higher in the RM group (13.5 ± 2.1 vs. 11.6 ± 3.1; *p* < .001). Operation time (40.3 ± 7.0 min vs. 143.1 ± 61.3 min;*p* < .001), intraparenchymal drainage catheter removal time (1.2 ± 0.4 vs. 2.1 ± 0.7 days; *p* < .001), and length of hospital stay (9.3 ± 2.7 vs. 11.1 ± 4.8 days; *p* = .013) were significantly shorter in the RM group. The incidence rates of pneumonia, gastrointestinal bleeding, and intracranial infection were significantly lower in the RM group. Although the incidence of rebleeding was lower in the RM group (1.3% vs. 5.4%), the difference was not significant. Six months after surgery, the Barthel Index, Glasgow Outcome Scale, and Karnofsky performance status scores were significantly higher, whereas the modified Rankin scale score was significantly lower in the RM group (*p* = .002, *p* = .007, *p* = .001, and *p* = .018, respectively). Two RM group patients (3.1%) and six CC group patients (12.2%) died between hospital discharge and 6 months after surgery (*p* = .127).

**Conclusion:**

The main advantages of robot‐assisted MISPT for supratentorial HICH were shown in minimally invasive, precision, and low incidences of complications. In addition, it may improve the prognosis significantly. Thus, it has great potential to be popularized and clinically applied in the future.

## Graphical abstract

Graphical Abstract: The main advantages of robot‐assisted MISPT for supratentorial HICH were shown in minimally invasive, precision, and low incidences of complications. In addition, it may improve the prognosis significantly. Thus, it has great potential to be popularized and clinically applied in the future.

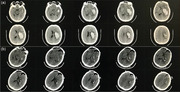

## 1 INTRODUCTION

Intracerebral hemorrhage (ICH) is associated with high rates of morbidity, disability, and mortality. It accounts for approximately 8%–30% of all strokes (Russell et al., 2006), depending on patient ethnicity and world region. ICH mortality is approximately 40% (Blacquiere et al., 2015; Morotti et al., 2016), which is two to six times higher than the mortality from ischemic stroke (Fayad & Awad, 1998). Stroke has become the leading cause of death and disability in China since 2015. In the 2020 data from the China Kadoorie Biobank, ICH accounted for 47% deaths from stroke in the first 28 days after stroke onset (Tu & Wang, 2023). The most common cause of ICH is arterial hypertension (Marquardt et al., 2005).

Surgical hematoma removal as an ICH treatment is being increasingly studied (De Oliveira Manoel, 2020; Kim et al., 2007; Miller et al., 2007; Tang et al., 2018). Recent trials of minimally invasive stereotactic puncture therapy (MISPT) in patients with hypertensive ICH (HICH) patients have reported good outcomes (Chen et al., 2017; De Oliveira Manoel et al., 2016; Hanley et al., 2016; Wartenberg & Mayer, 2015). This therapy can remove hematoma in a relatively short period of time to potentially enhance neurological recovery while minimizing brain injury and complications (Ramanan & Shankar, 2013; Staykov et al., 2010; Wang et al., 2021). However, compared with conventional craniotomy (CC), frame‐assisted MISPT had some disadvantages, such as inability to stop bleeding under direct vision, susceptibility to secondary bleeding, and unclear timing of surgical intervention. Besides, it was more suitable in treating patients with small‐volume hematoma (Han et al., 2017). Kim et al. (2019) reported that treatment of large‐volume spontaneous ICH (≥50 mL) using frame‐assisted followed by thrombolysis is feasible and associated with a low complication rate. Robot‐assisted MISPT is a recent development in ICH treatment that uses stereotactic and artificial intelligence technology (Alan et al., 2017). We have previously studied robot‐assisted MISPT for treating HICH and confirmed its superiority to the frame‐assisted approach (Xiao et al., 2018). Therefore, in the present study, we compared the efficacy of robot‐assisted MISPT and CC for supratentorial HICH and evaluated whether robot‐assisted MISPT could solve the shortcomings mentioned before.

## 2 MATERIALS AND METHODS

### 2.1 Study design and setting

We retrospectively reviewed 413 patients with HICH in Nanyang Central Hospital affiliated with Zhengzhou University from January to December 2020. Inclusion criteria were as follows: (1) history of hypertension or hypertension recorded at hospital admission, (2) supratentorial hematoma, (3) hematoma volume 20–80 mL, (4) age 30–70 years, (5) preoperative Glasgow Coma scale (GCS) score ≥5, and (6) hemorrhage onset within 24 h of presentation. Some patients underwent computed tomography angiography (CTA) to exclude an underlying vascular abnormality. We excluded patients with intraventricular hemorrhage; clotting dysfunction or platelet abnormality caused by disease or pharmaceutical use; cerebral herniation; systemic or intracranial infection; expected survival <6 months; and ICH caused by aneurysm, venous malformation, tumor, trauma, or other identifiable cause. Pregnant women and patients with multiple hemorrhages were also excluded. The study was approved by the ethics committee of Nanyang Central Hospital affiliated with Zhengzhou University (IRB No. 21‐2A‐15).

### 2.2 Methods

#### 2.2.1 Robot‐assisted MISPT(RM group)

**FIGURE 1:**
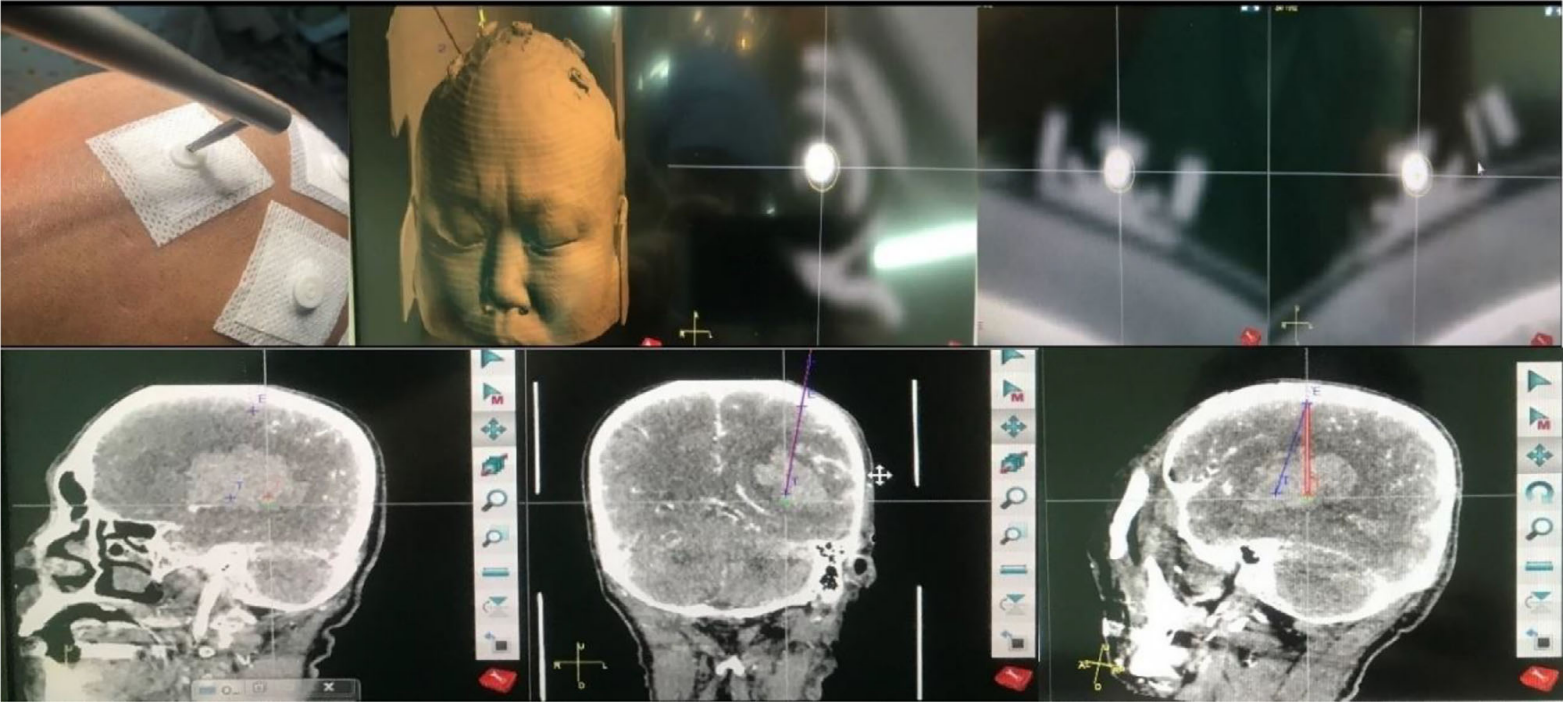
A surgical procedure designed to avoid critical functional areas and blood vessels.

Robot‐assisted MISPT procedures were performed using the ROSA system (Zimmer Biomet). The patient was sedated using diazepam, and the operation was performed under local anesthesia. Head CTA was performed after five scalp markers were placed on the patient's head. Imaging data were transmitted to the ROSA robot system computer workstation, and the surgical plan was designed. The target and trajectory for drainage tube placement were selected, carefully avoiding the ventricles, critical brain structures, and blood vessels. In general, the hematoma center was selected as the target. The patient's head was then affixed to a Fisher head frame (Leibinger), which was then connected to the operating bed. The five scalp markers were registered into the image‐guidance system (Figure 1) and the entry point was selected under robot laser guidance to determine the incision site. After skin disinfection and draping, the scalp was incised, and a 1 cm burr hole was placed in the skull using a drill. The dura was incised (diameter, 3 mm), and the robot arm automatically moved according to the surgical plan. The puncture tube (diameter, 1.5 mm) was placed under robot arm guidance. The hematoma was gently suctioned and irrigated repeatedly with saline until the fluid was clear. After aspiration and irrigation, a drainage tube was placed in the hematoma center.

#### 2.2.2 CC (CC group)

In patients undergoing CC, a small skull‐window craniotomy was performed under general anesthesia to remove the hematoma. Whether the bone flap was removed or not depends on the specific situation. Brain tissue was protected as much as possible during surgery, and the hematoma was removed. After the operation, a drainage tube was placed in the hematoma center.

#### 2.2.3 Postoperative management

All patients were transferred to the neurosurgery intensive care unit after surgery, where they stayed until their condition was stable and appropriate for the general ward. Patients were treated in accordance with the American Heart Association/Stroke Association Stroke Council spontaneous ICH treatment guidelines (Hemphill et al., 2015).

Head CT was performed to measure the postoperative hematoma volume and observe the place of drainage tube 2 h after the operation and then daily until discharge. Blood pressure was strictly maintained within normal range. Intracranial pressure was measured via lumbar puncture 3 days after surgery. A lumbar drain was placed if the hematoma entered the ventricle. After surgery, intracavitary urokinase was injected through the drainage tube based on residual hematoma volume. Based on our experience, the urokinase dose administered was 30,000 U. After injection, the drainage tube was closed for 2 h. Repeat injections were performed as needed, based on subsequent head CT results. The drainage tube was removed when hematoma volume was <3 mL or on day 7 after surgery.

#### 2.2.4 Study data

Patient demographics and clinical characteristics were recorded. Hematoma volume before and after surgery was calculated using the Tada formula (*abc*/2, where *a* is the largest diameter of the hematoma on axial images, *b* is the largest diameter perpendicular to *a* on the same image slice, and *c* is the number of slices in which the hematoma is visualized multiplied by the slice thickness) (Kothari et al., 1996). Slice thickness was set to 1 cm. The primary outcome was the postoperative hematoma volume at discharge. The secondary outcomes were the GCS score at discharge, operation time, postoperative timing of intraparenchymal drainage catheter removal, length of hospital stay, complications, mortality, and 6‐month postoperative functional scores including the Glasgow Outcome Scale (GOS) score, Barthel Index (BI) score, modified Rankin scale (MRS) score, and Karnofsky performance scale (KPS) score.

Complications, including pneumonia, gastrointestinal bleeding, intracranial infection, and rebleeding, were evaluated during hospitalization. Rebleeding was defined as a volume growth of >6 mL or 33% (Dowlatshahi et al., 2011) since the last CT scan as assessed by two experienced neurosurgeons. Mortality was evaluated early (7 days after surgery or at discharge) and at the 6‐month follow‐up. After discharge, the patients were followed up by a research coordinator.

### 2.3 Statistical analysis

Statistical analyses were performed using SPSS software version 24.0 (IBM Corp.). Categorical data are expressed as numbers with percentage and were compared using the *χ* ^2^ test or Fisher's exact test. Continuous data with a normal distribution are expressed as means with standard deviation and were compared using the independent sample *t*‐test. All tests were two‐sided. *p* < .05 was considered significant.

## 3 RESULTS

### 3.1 Patient characteristics

**FIGURE 2:**
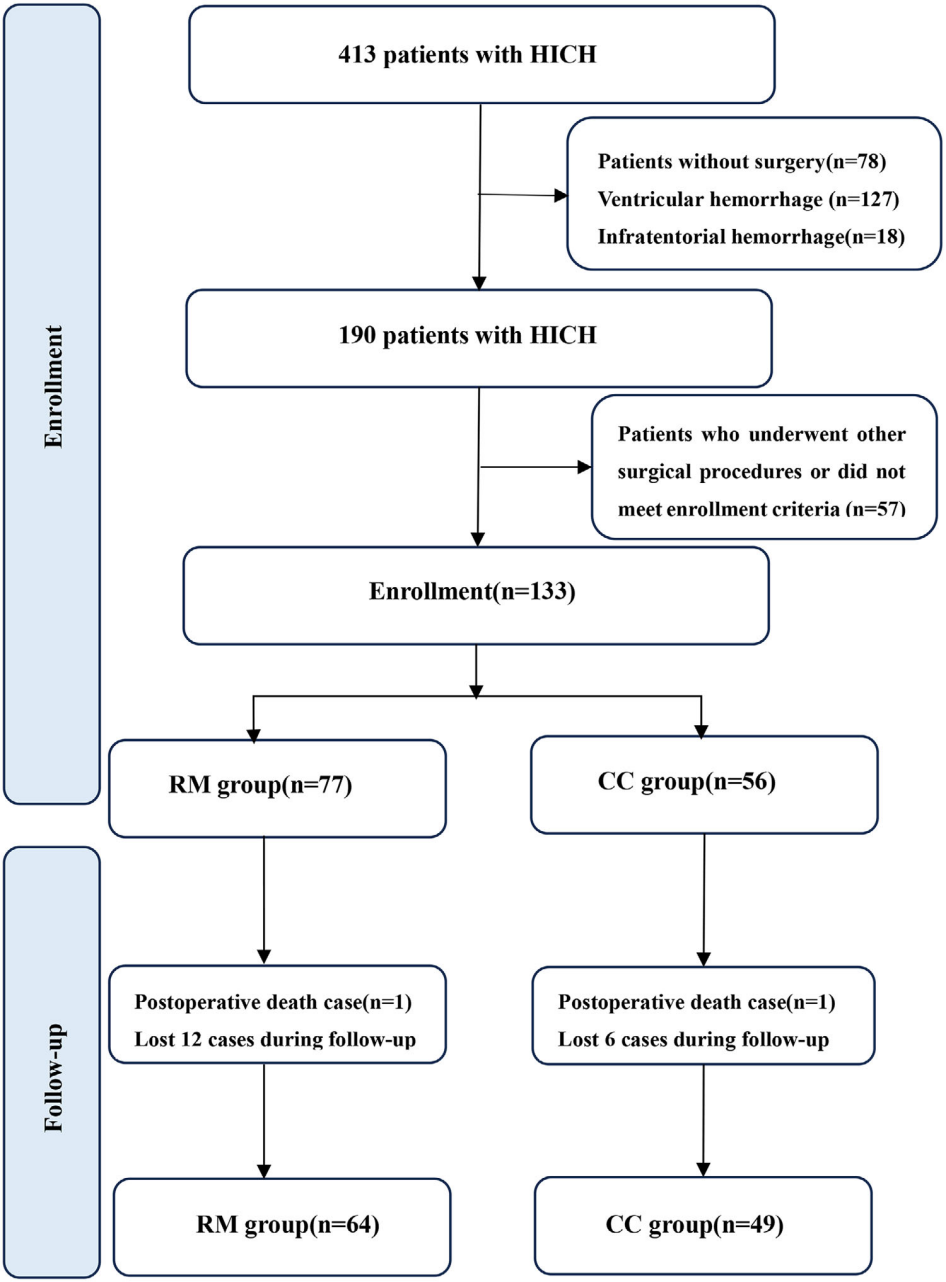
Study flowchart.

A total of 133 patients were included for analysis: 77 patients who underwent robot‐assisted MISPT and 56 who underwent CC. A study flowchart is shown in Figure 2. Baseline patient characteristics, including age, sex, blood pressure, history of hypertension, GCS score, and hematoma volume, location, and laterality, are shown in Table 1.

**TABLE 1:** Patient characteristics according to group.

Group	RM	CC	*t*/*χ* ^2^	*p*
Number of people (number)	77	56		
Gender (male/female)	54/23	38/18	0.079	0.779
Age (year)	52.8 ± 9.6	55.3 ± 7.8	−1.648	0.102
Blood pressure (mmHg)				
Systolic pressure	167.3 ± 22.1	169.7 ± 23.3	−0.603	0.548
Diastolic pressure	98.9 ± 13.1	99.2 ± 16.6	−0.110	0.913
Hypertension history (year)	5.6 ± 4.6	5.0 ± 4.4	0.766	0.445
GCS score Hematoma volume (mL)	10.7 ± 2.2 38.4 ± 10.4	9.8 ± 2.8 41.1 ± 11.0	1.868 −1.427	0.065 0.156
Hematoma volume (*n*/%)			3.881	0.144
20–30	22/28.6	8/14.3		
31–50	47/61.0	42/75.0		
51–80	8/10.4	6/10.7		
Hematoma location (*n*/%)			0.698	0.705
Basal ganglia	29/37.7	25/44.6		
Thalamus	33/42.9	22/39.3		
Lobar	15/19.5	9/16.1		
Hematoma direction (*n*/%)			2.930	0.087
Left	38/49.4	36/64.3		
Right	39/50.6	20/35.7		

### 3.2 Clinical results

Table 2 shows the postoperative hematoma volume and GCS score data as well as postoperative complications according to group. Other early outcome indicators are shown in Table 3. Hematoma volume at discharge did not significantly differ between the groups (2.6 ± 2.1 and 2.4 ± 2.1 mL, respectively). GCS score (13.5 ± 2.1 vs. 11.6 ± 3.1; *p* < .001), operation time (40.3 ± 7.0 vs. 143.1 ± 61.3 min; *p* < .001), postoperative intraparenchymal drainage catheter removal time (1.2 ± 0.4 vs. 2.1 ± 0.7 days; *p* < .001), and length of hospital stay (9.3 ± 2.7 vs. 11.1 ± 4.8 days; *p* = .013) significantly differed between the RM and CC groups. Besides, incidence rates for postoperative pneumonia, gastrointestinal bleeding, and intracranial infection were significantly lower in the RM group (*p* = .001, *p* < .001, and *p* = .027, respectively). One patient in the RM group died of pneumonia after surgery; one CC group patient died of old age and frailty. Incidence of rebleeding and early mortality did not significantly differ between the groups.

**TABLE 2:** Comparison of primary efficacy indicator and postoperative GCS score and complications.

Group	RM	CC	*t*/*χ* ^2^	*p*
Number of people (number)	77	56		
Hematoma volume (mL)				
At discharge	2.6 ± 2.1	2.4 ± 2.1	0.573	0.568
GCS score				
At discharge	13.5 ± 2.1	11.6 ± 3.1	4.136	< 0.001
Incidence of postoperative complications (*n*/%)				
Pneumonia	27/35.1	36/64.3	11.104	0.001
Gastrointestinal bleeding	7/9.1	20/35.7	14.203	< 0.001
Intracranial infection	0/0	5/8.9	4.889	0.027
Incidence of rebleeding	1/1.3	3/5.4	0.704	0.402

**TABLE 3:** Comparison of other secondary efficacy indicators and early mortality.

Group	RM	CC	*t*/*χ* ^2^	*p*
Operation time (min)	40.3 ± 7.0	143.1 ± 61.3	−12.476	< 0.001
Drainage catheter removal time (day)	1.2 ± 0.4	2.1 ± 0.7	−8.374	< 0.001
Postoperative hospitalization time (day) Mortality (*n*/%)	9.3 ± 2.7 1/1.3	11.1 ± 4.8 1/1.8	−2.538 —	0.013 1.000

### 3.3 Six‐month follow‐up

Table 4 shows the 6‐month follow‐up indicators according to group. Eighteen patients were lost to follow‐up (13.7%). Two RM group patients (3.1%) and six CC group patients (12.2%) died between hospital discharge and 6 months after surgery (*p* = .127); among these, one died of renal failure at 2 months. GOS score (3.3 ± 0.7 vs. 3.0 ± 0.6; *p* = .007), BI score (54.0 ± 20.9 vs. 40.9 ± 21.1; *p* = .002), KPS score (55.2 ± 14.6 vs. 47.0 ± 9.9; *p* = .001), and MRS score (2.9 ± 0.9 vs. 3.3 ± 0.8; *p* = .018) at 6 months significantly differed between the groups.

**TABLE 4:** Comparison of indicators at the 6‐month follow‐up according to group.

Group	RM	CC	t/χ^2^	*p*
Number of people (number) BI	64 54.0 ± 20.9	49 40.9 ± 21.1	3.128	0.002
GOS	3.3 ± 0.7	3.0 ± 0.6	2.729	0.007
KPS	55.2 ± 14.6	47.0 ± 9.9	3.429	0.001
MRS	2.9 ± 0.9	3.3 ± 0.8	−2.414	0.018
Mortality (*n*/%)	2/3.1	6/12.2	2.331	0.127

## 4 DISCUSSION

In this retrospective analysis, we found that robot‐assisted MISPT in patients with supratentorial HICH could effectively remove the hematoma, promote recovery of consciousness, and shorten the length of hospital stay with a low incidence of complications. The 6‐month clinical outcomes were better in patients who underwent robot‐assisted MISPT than in those who underwent CC. Changing the surgical method or surgical strategy provides more choices for the timing of robot‐assisted minimally invasive surgery.

HICH directly causes brain injury, intracranial hypertension, and sometimes cerebral herniation (Sangha & Gonzales, 2011). Edema forms around the site of hemorrhage, which causes an increase in local pressure (Zhou et al., 2011). In addition, thrombin in the hemorrhaged blood causes secondary brain parenchyma damage because of its neurotoxic effects (Xi et al., 2006). The objectives of surgical intervention are to reduce hematoma volume, prevent further bleeding, reduce mass effect and intracranial pressure, maintain cerebrospinal fluid circulation, and prevent secondary neurological deterioration by reducing local ischemia and removing toxic chemicals (Keep et al., 2005; Siddique et al., 2000; Xi et al., 2006).

CC for treatment of HICH provides a good exposure of the surgical field, enables rapid removal of hematoma, alleviates cerebral edema, improves cerebrospinal fluid circulation, achieves hemostasis, and reduces intracranial pressure. However, the operation time is long, and it is associated with risks of brain injury, infection, and other complications (Brouwers & Goldstein, 2011; Hwang et al., 2008; Salazar et al., 1986).

Precise and less invasive treatments are two current trends in neurosurgery. MISPT is associated with less brain tissue injury, less bleeding, milder cerebral edema, shorter operation time and hospital stay, and faster recovery (Li et al., 2017). Frame‐assisted MISPT is particularly suitable for the removal of deep hematomas and elderly and infirm patients who cannot tolerate craniotomy because of its less invasive nature (Matsumoto & Hondo, 1984; Wang et al., 2019). However, preoperative preparations for frame‐assisted MISPT are complex and time‐consuming; moreover, it is associated with a relatively high infection rate, does not avoid blood vessels, and is associated with a high incidence of rebleeding (Xiao et al., 2018). Avoidance of critical brain structures and blood vessels has always been of great importance to successful brain surgery. Today, robot‐assisted MISPT is another treatment option.

The ROSA system enables preoperative planning and precise positioning, is simple to use, and has a wide range of applications (Hoshide et al., 2016). It is also accurate and has a variety of registration modes. Bone markers are the gold standard for stereotactic procedures, as they are not influenced by skin shift during imaging and registration (Mascott et al., 2006). Accordingly, they are preferentially used in robot‐assisted deep brain stimulation procedures (Jin et al., 2020). We used scalp markers for registration because they are more convenient and precise enough for treatment of HICH. The surgeon can control the surgical accuracy within 2 mm (Xiao et al., 2018), which is key from a safety perspective. In contrast, the systematic error for frame‐assisted stereotactic procedures ranges from 1.0 to 5.2 mm (Widmann et al., 2009).

**FIGURE 3:**
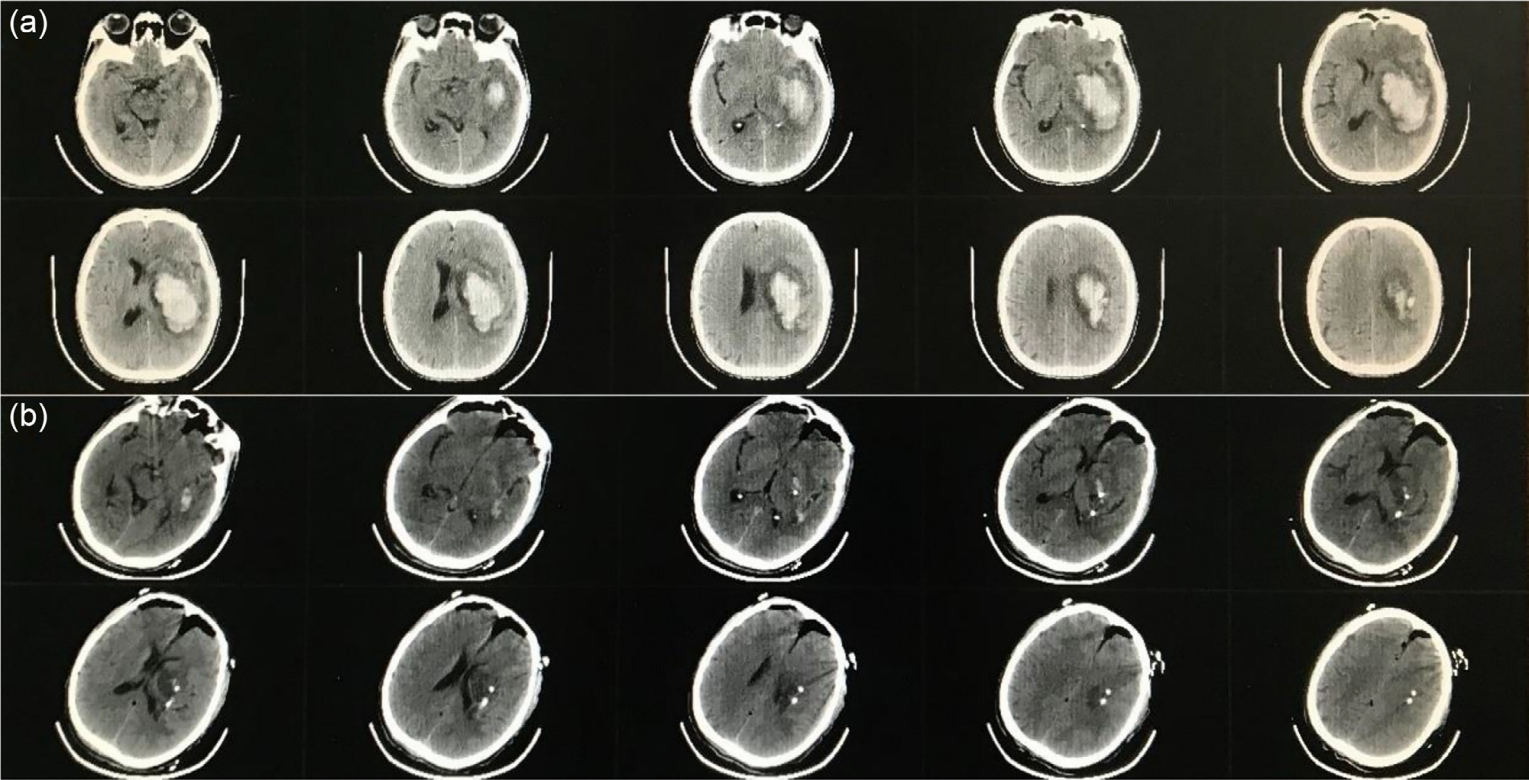
Head computed tomography (A) before and (B) after robot‐assisted minimally invasive stereotactic puncture therapy.

**FIGURE 4:**
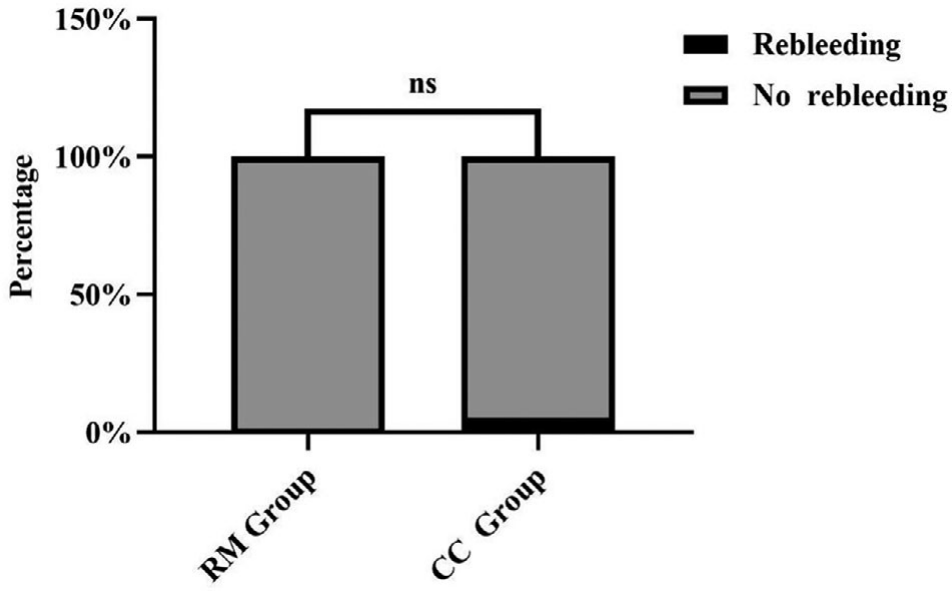
Rebleeding incidence after surgery.

In our study, hematoma volume at discharge did not significantly differ between the RM and CC groups. Figure 3 shows pre‐ and postoperative CT imaging from a patient in the RM group. Compared with CC, robot‐assisted MISPT requires a smaller incision, has a shorter operation time, and causes less brain tissue injury. Operation time was much shorter in the RM group (40.3 ± 7.0 vs. 143.1 ± 61.3 min), as MISPT has a shorter preparation time and was performed under local anesthesia. Area of brain exposure is lower with MISPT, which reduced the incidence of postoperative intracranial infection (0% vs. 8.9%). CC is more traumatic to the patient and may cause a strong stress reaction, which might explain the higher rate of gastrointestinal bleeding in the CC group (35.7% vs. 9.1%). The incidence of postoperative pneumonia was significantly higher in the CC group as well (64.3% vs. 35.1%). Postoperative GCS score was significantly higher in the RM group (13.5 ± 2.1 vs. 11.6 ± 3.1), suggesting that robot‐assisted MISPT is better than CC in promoting neurological recovery and protecting brain tissue. During hospitalization, one patient in the CC group died of old age and frailty, and one RM group patient died of lung infection, possibly related to heart disease. Surgical planning for robot‐assisted MISPT can be freely designed according to hematoma morphology, allowing access via a path that avoids functional areas and blood vessels (Han et al., 2017; Xiao et al., 2018). In a previous study of frame‐assisted MISPT in ICH, the incidence of rebleeding was 1.6% (Marquardt et al., 2003), which is comparable to our robot‐assisted MISPT rebleeding rate (1.3%). The ability of robot‐assisted MISPT to avoid blood vessels should reduce the incidence of rebleeding; we found no significant difference with the CC group rebleeding rate (5.4%) (Figure 4). Postoperative intraparenchymal drainage catheter removal time and length of hospital stay were significantly lower in the RM group. At the same time, BI, GOS, KPS, and MRS scores were better, suggesting that robot‐assisted MISPT has many advantages over CC in treating HICH.

The optimal timing of MISPT for HICH surgery is controversial. Some scholars reported that the risk of HICH rebleeding is high within the first 6 h of hemorrhage (Qureshi et al., 2009): Some operations performed in the first 6 h might be necessary to consider the risk of rebleeding. We typically wait 3 days, which seems to achieve satisfactory results (Han et al., 2017). Both approaches were applicable in the patients selected in our study. In case the condition of patients deteriorated and the robot‐assisted MISPT was performed, only approximately half of the hematoma volume was aspirated during surgery. In addition, the remaining volume was treated with intracavitary urokinase injection and drainage via a catheter. Through this approach, the trouble caused by unclear timing of surgery to the surgeons and the shortcoming of frame‐assisted MISPT being unable to stop bleeding under direct vision and prone to rebleeding can be solved to some extent.

This study has several limitations, including its small sample size and retrospective design. In addition, factors such as the accuracy of the surgical robot, the time of diagnosis, and the unclear optimal timing may have affected the results to some extent. Patient follow‐up was relatively short, and the long‐term clinical efficacy of the treatment is therefore unknown. A multicenter randomized controlled study in terms of the large sample size, the timing of surgery, and the long‐term follow‐up is needed to further examine the surgical treatment of HICH.

## 5 CONCLUSIONS

The main advantages of robot‐assisted MISPT for supratentorial HICH were shown in minimally invasive, precision, and low incidences of complications. In addition, it may improve the prognosis significantly. Thus, it has great potential to be popularized and clinically applied in the future.

## AUTHOR CONTRIBUTIONS

**Weiyi Han**: Writing—original draft. **Aotan Xie**: Data curation. **Taoli Chen**: Investigation. **Xiao Sun**: Software. **Xianzhi Liu**: Project administration.

## CONFLICT OF INTEREST STATEMENT

The authors declared that there are no conflicts of interest.

### PEER REVIEW

The peer review history for this article is available at https://publons.com/publon/10.1002/brb3.3402.

## Data Availability

The data that support the findings of this study are available from the corresponding author upon reasonable request.

## References

[brb33402-bib-0001] Alan, N. , Lee, P. , Ozpinar, A. , Gross, B. A. , & Jankowitz, B. T. (2017). Robotic stereotactic assistance (ROSA) utilization for minimally invasive placement of intraparenchymal hematoma and intraventricular catheters: Case report. World Neurosurgery, 108, 996.e7–996.e10. 10.1016/j.wneu.2017.09.027 28919568

[brb33402-bib-0002] Blacquiere, D. , Demchuk, A. M. , Al‐Hazzaa, M. , Deshpande, A. , Petrcich, W. , Aviv, R. I. , Rodriguez‐Luna, D. , Molina, C. A. , Silva Blas, Y. , Dzialowski, I. , Czlonkowska, A. , Boulanger, J.‐M. , Lum, C. , Gubitz, G. , Padma, V. , Roy, J. , Kase, C. S. , Bhatia, R. , Hill, M. D. , … Dowlatshahi, D. (2015). Intracerebral hematoma morphologic appearance on noncontrast computed tomography predicts significant hematoma expansion. Stroke; A Journal of Cerebral Circulation, 46(11), 3111–3116. 10.1161/STROKEAHA.115.010566 26451019

[brb33402-bib-0003] Brouwers, H. B. , & Goldstein, J. N. (2011). Therapeutic strategies in acute intracerebral hemorrhage. Neurotherapeutics, 9(1), 87–98. 10.1007/s13311-011-0091-8 PMC3271150 22139592

[brb33402-bib-0004] Chen, S. , Zhao, B. , Wang, W. , Shi, L. , Reis, C. , & Zhang, J. (2017). Predictors of hematoma expansion predictors after intracerebral hemorrhage. Oncotarget, 8(51), 89348–89363. 10.18632/oncotarget.19366 29179524 PMC5687694

[brb33402-bib-0005] De Oliveira Manoel, A. L. (2020). Surgery for spontaneous intracerebral hemorrhage. Critical Care (London, England), 24(1), 45. 10.1186/s13054-020-2749-2 32033578 PMC7006102

[brb33402-bib-0006] De Oliveira Manoel, A. L. , Goffi, A. , Zampieri, F. G. , Turkel‐Parrella, D. , Duggal, A. , Marotta, T. R. , Macdonald, R. L. , & Abrahamson, S. (2016). The critical care management of spontaneous intracranial hemorrhage: A contemporary review. Critical Care (London, England), 20, 272. 10.1186/s13054-016-1432-0 27640182 PMC5027096

[brb33402-bib-0007] Dowlatshahi, D. , Demchuk, A. M. , Flaherty, M. L. , Ali, M. , Lyden, P. L. , & Smith, E. E. (2011). Defining hematoma expansion in intracerebral hemorrhage: Relationship with patient outcomes. Neurology, 76(14), 1238–1244. 10.1212/WNL.0b013e3182143317 21346218 PMC3068004

[brb33402-bib-0008] Fayad, P. B. , & Awad, I. A. (1998). Surgery for intracerebral hemorrhage. *Neurology*, *51*(3 Suppl 3), S69 –S73. 10.1212/wnl.51.3_suppl_3.s69 9744840

[brb33402-bib-0009] Han, W. Y. , Tao, Y. Q. , Xu, F. , Zhang, Y. Q. , Li, Z. Y. , & Liang, G. B. (2017). The short‐ and long‐term efficacy analysis of stereotactic surgery combined external ventricular drainage in the treatment of the secondary intraventricular hemorrhage. Brain and Behavior, 7(12), e00864. 10.1002/brb3.864 29299383 PMC5745243

[brb33402-bib-0010] Hanley, D. F. , Thompson, R. E. , Muschelli, J. , Rosenblum, M. , Mcbee, N. , Lane, K. , Bistran‐Hall, A. J. , Mayo, S. W. , Keyl, P. , Gandhi, D. , Morgan, T. C. , Ullman, N. , Mould, W. A. , Carhuapoma, J. R. , Kase, C. , Ziai, W. , Thompson, C. B. , Yenokyan, G. , Huang, E. , … Zuccarello, M. (2016). Safety and efficacy of minimally invasive surgery plus alteplase in intracerebral haemorrhage evacuation (MISTIE): A randomised, controlled, open‐label, phase 2 trial. Lancet Neurology, 15(12), 1228–1237. 10.1016/S1474-4422(16)30234-4 27751554 PMC5154627

[brb33402-bib-0011] Hemphill, J. C. , Greenberg, S. M. , Anderson, C. S. , Becker, K. , Bendok, B. R. , Cushman, M. , Fung, G. L. , Goldstein, J. N. , Macdonald, R. L. , Mitchell, P. H. , Scott, P. A. , Selim, M. H. , & Woo, D. (2015). Guidelines for the management of spontaneous intracerebral hemorrhage: A guideline for healthcare professionals from the American Heart Association/American Stroke Association. Stroke; A Journal of Cerebral Circulation, 46(7), 2032–2060. 10.1161/STR.0000000000000069 26022637

[brb33402-bib-0012] Hoshide, R. , Calayag, M. , Meltzer, H. S. , Levy, M. L. , & Gonda, D. D. (2016). 352 Robot‐assisted endoscopic third ventriculostomy. Neurosurgery, 63(Suppl 1), 204. 10.1227/01.neu.0000489841.88045.a3 28598265

[brb33402-bib-0013] Hwang, J. H. , Han, J. W. , Park, K. B. , Lee, C. H. , Park, I. S. , & Jung, J.‐M. (2008). Stereotactic multiplanar reformatted computed tomography‐guided catheter placement and thrombolysis of spontaneous intracerebral hematomas. Journal of Korean Neurosurgical Society, 44(4), 185–189. 10.3340/jkns.2008.44.4.185 19096674 PMC2588315

[brb33402-bib-0014] Jin, H. , Gong, S. , Tao, Y. , Huo, H. , Sun, X. , Song, D. , Xu, M. , Xu, Z. , Liu, Y. , Wang, S. , Yuan, L. , Wang, T. , Song, W. , & Pan, H. (2020). A comparative study of asleep and awake deep brain stimulation robot‐assisted surgery for Parkinson's disease. NPJ Parkinson's Disease, 6, 27. 10.1038/s41531-020-00130-1 PMC7536209 33083521

[brb33402-bib-0016] Keep, R. F. , Xi, G. , Hua, Y. , & Hoff, J. T. (2005). The deleterious or beneficial effects of different agents in intracerebral hemorrhage. Stroke; A Journal of Cerebral Circulation, 36(7), 1594–1596. 10.1161/01.STR.0000170701.41507.e1 15933250

[brb33402-bib-0017] Kim, C. H. , Choi, J. H. , & Park, H. S. (2019). Safety and efficacy of minimally invasive stereotactic aspiration with multicatheter insertion compared with conventional craniotomy for large spontaneous intracerebral hemorrhage (>/= 50 mL). World Neurosurgery, 128, e787–ee795. 31078808 10.1016/j.wneu.2019.04.258

[brb33402-bib-0018] Kim, I.‐S. , Son, B.‐C. , Lee, S.‐W. , Sung, J.‐H. , & Hong, J.‐T. (2007). Comparison of frame‐based and frameless stereotactic hematoma puncture and subsequent fibrinolytic therapy for the treatment of supratentorial deep seated spontaneous intracerebral hemorrhage. Minimally Invasive Neurosurgery, 50(2), 86–90. 10.1055/s-2007-982503 17674294

[brb33402-bib-0019] Kothari, R. U. , Brott, T. , Broderick, J. P. , Barsan, W. G. , Sauerbeck, L. R. , Zuccarello, M. , & Khoury, J. (1996). The ABCs of measuring intracerebral hemorrhage volumes. Stroke; A Journal of Cerebral Circulation, 27(8), 1304–1305. 10.1161/01.STR.27.8.1304 8711791

[brb33402-bib-0020] Li, Y. , Yang, R. , Li, Z. , Yang, Y. , Tian, B. , Zhang, X. , Wang, B. , Lu, D. , Guo, S. , Man, M. , Yang, Y. , Luo, T. , Gao, G. , & Li, L. (2017). Surgical evacuation of spontaneous supratentorial lobar intracerebral hemorrhage: Comparison of safety and efficacy of stereotactic aspiration, endoscopic surgery, and craniotomy. World Neurosurgery, 105, 332–340. 10.1016/j.wneu.2017.05.134 28578111

[brb33402-bib-0021] Marquardt, G. , Wolff, R. , Janzen, R. W. , & Seifert, V. (2005). Basal ganglia haematomas in non‐comatose patients: Subacute stereotactic aspiration improves long‐term outcome in comparison to purely medical treatment. Neurosurgical Review, 28(1), 64–69. 15455261 10.1007/s10143-004-0355-4

[brb33402-bib-0022] Marquardt, G. , Wolff, R. , & Seifert, V. (2003). Multiple target aspiration technique for subacute stereotactic aspiration of hematomas within the basal ganglia. Surgical Neurology, 60(1), 8–13. 10.1016/S0090-3019(03)00084-3 12865001

[brb33402-bib-0023] Mascott, C. R. , Sol, J. C. , Bousquet, P. , Lagarrigue, J. , Lazorthes, Y. , & Lauwers‐Cances, V. (2006). Quantification of true in vivo (application) accuracy in cranial image‐guided surgery: Influence of mode of patient registration. *Neurosurgery*, *59*(1 Suppl 1), ONS146–ONS156. 10.1227/01.NEU.0000220089.39533.4E 16888546

[brb33402-bib-0024] Matsumoto, K. , & Hondo, H. (1984). CT‐guided stereotaxic evacuation of hypertensive intracerebral hematomas. Journal of Neurosurgery, 61(3), 440–448. 10.3171/jns.1984.61.3.0440 6379125

[brb33402-bib-0025] Miller, C. M. , Vespa, P. M. , Mcarthur, D. L. , Hirt, D. , & Etchepare, M. (2007). Frameless stereotactic aspiration and thrombolysis of deep intracerebral hemorrhage is associated with reduced levels of extracellular cerebral glutamate and unchanged lactate pyruvate ratios. Neurocrit Care, 6(1), 22–29. 10.1385/NCC:6:1:22 17356187

[brb33402-bib-0026] Morotti, A. , Jessel, M. J. , Brouwers, H. B. , Falcone, G. J. , Schwab, K. , Ayres, A. M. , Vashkevich, A. , Anderson, C. D. , Viswanathan, A. , Greenberg, S. M. , Gurol, M. E. , Romero, J. M. , Rosand, J. , & Goldstein, J. N. (2016). CT angiography spot sign, hematoma expansion, and outcome in primary pontine intracerebral hemorrhage. Neurocrit Care, 25(1), 79–85. 10.1007/s12028-016-0241-2 26759226 PMC4940347

[brb33402-bib-0027] Qureshi, A. I. , Mendelow, A. D. , & Hanley, D. F. (2009). Intracerebral haemorrhage. Lancet, 373(9675), 1632–1644. 10.1016/S0140-6736(09)60371-8 19427958 PMC3138486

[brb33402-bib-0028] Ramanan, M. , & Shankar, A. (2013). Minimally invasive surgery for primary supratentorial intracerebral haemorrhage. Journal of Clinical Neuroscience, 20(12), 1650–1658. 10.1016/j.jocn.2013.03.022 24161339

[brb33402-bib-0029] Russell, M. W. , Boulanger, L. , Joshi, A. V. , Neumann, P. J. , & Menzin, J. (2006). The economic burden of intracerebral hemorrhage: Evidence from managed care. Managed Care Interface, 19(6), 24–28. 34. 16892657

[brb33402-bib-0030] Salazar, J. , Vaquero, J. , Martinez, P. , Santos, H. , Martinez, R. , & Bravo, G. (1986). Clinical and CT scan assessment of benign versus fatal spontaneous cerebellar haematomas. Acta Neurochirurgica, 79(2–4), 80–86. 10.1007/BF01407449 PMID: 3962747 3962747

[brb33402-bib-0031] Sangha, N. , & Gonzales, N. R. (2011). Treatment targets in intracerebral hemorrhage. Neurotherapeutics, 8(3), 374–387. 10.1007/s13311-011-0055-z 21732225 PMC3250268

[brb33402-bib-0032] Siddique, M. S. , Fernandes, H. M. , Arene, N. U. , Wooldridge, T. D. , Fenwick, J. D. , & Mendelow, A. D. (2000). Changes in cerebral blood flow as measured by HMPAO SPECT in patients following spontaneous intracerebral haemorrhage. Acta Neurochirurgica Supplement, 76, 517–520. 11450081 10.1007/978-3-7091-6346-7_108

[brb33402-bib-0033] Staykov, D. , Huttner, H. B. , Köhrmann, M. , Bardutzky, J. , & Schellinger, P. D. (2010). Novel approaches to the treatment of intracerebral haemorrhage. International Journal of Stroke, 5(6), 457–465. 10.1111/j.1747-4949.2010.00487.x 21050402

[brb33402-bib-0034] Tang, Y. , Yin, F. , Fu, D. , Gao, X. , Lv, Z. , & Li, X. (2018). Efficacy and safety of minimal invasive surgery treatment in hypertensive intracerebral hemorrhage: A systematic review and meta‐analysis. BMC Neurology [Electronic Resource], 18(1), 136. 10.1186/s12883-018-1138-9 30176811 PMC6120062

[brb33402-bib-0035] Tu, W. J. , & Wang, L. D. (2023). Special writing group of china stroke surveillance report. China stroke surveillance report 2021. Military Medical Research, 10(1), 33. 37468952 10.1186/s40779-023-00463-x PMC10355019

[brb33402-bib-0036] Wang, L. , Zhang, L. , Mao, Y. , Li, Y. , Wu, G. , & Li, Q. (2021). Regular‐shaped hematomas predict a favorable outcome in patients with hypertensive intracerebral hemorrhage following stereotactic minimally invasive surgery. Neurocrit Care, 34(1), 259–270. 10.1007/s12028-020-00996-2 32462410

[brb33402-bib-0037] Wang, Y. , Jin, H. , Gong, S. , Yang, X. , Sun, X. , Xu, M. , Liu, Y. , Wang, S. , Song, W. , & Tao, Y. (2019). Efficacy analysis of robot‐assisted minimally invasive surgery for small‐volume spontaneous thalamic hemorrhage. World Neurosurgery, 131, e543–e549. 10.1016/j.wneu.2019.07.224 31398520

[brb33402-bib-0038] Wartenberg, K. E. , & Mayer, S. A. (2015). Ultra‐early hemostatic therapy for intracerebral hemorrhage: Future directions. Frontiers of Neurology and Neuroscience, 37, 107–129. 10.1159/000437117 26588167

[brb33402-bib-0039] Widmann, G. , Stoffner, R. , Sieb, M. , & Bale, R. (2009). Target registration and target positioning errors in computer‐assisted neurosurgery: Proposal for a standardized reporting of error assessment. The International Journal of Medical Robotics, 5(4), 355–365. 10.1002/rcs.271 19565464

[brb33402-bib-0040] Xi, G. , Keep, R. F. , & Hoff, J. T. (2006). Mechanisms of brain injury after intracerebral haemorrhage. Lancet Neurology, 5, 53–63. 10.1016/S1474-4422(05)70283-0 16361023

[brb33402-bib-0041] Xiao, S. , Yingqun, T. , Hai, J. , Feng, X. , Xiaoqiu, L. , Xinhong, W. , Xingwang, Y. , Yun, W. , Mengting, X. , & Junhe, W. (2018). Comparative study of surgical treatment of hypertensive intracerebral hemorrhage assisted with ROSA system and stereotactic frame. Chinese Journal of Neurosurgery, 34, 674–677.

[brb33402-bib-0042] Zhou, H. , Zhang, Y. , Liu, L. , Han, X. , Tao, Y. , Tang, Y. , Hua, W. , Xue, J. , & Dong, Q. (2011). A prospective controlled study: Minimally invasive stereotactic puncture therapy versus conventional craniotomy in the treatment of acute intracerebral hemorrhage. BMC Neurology [Electronic Resource], 11, 76. 10.1186/1471-2377-11-76 21699716 PMC3142495

